# Modular Microfluidics: Current Status and Future Prospects

**DOI:** 10.3390/mi13081363

**Published:** 2022-08-22

**Authors:** Xiaochen Lai, Mingpeng Yang, Hao Wu, Dachao Li

**Affiliations:** 1 School of Automation, Nanjing University of Information Science & Technology, Nanjing 210044, China; 2 State Key Laboratory of Precision Measuring Technology and Instruments, Tianjin University, Tianjin 300072, China

**Keywords:** modular microfluidics, reconfiguration, reusability, on-demand deployment, rapid prototyping, organs-on-a-chip

## Abstract

This review mainly studies the development status, limitations, and future directions of modular microfluidic systems. Microfluidic technology is an important tool platform for scientific research and plays an important role in various fields. With the continuous development of microfluidic applications, conventional monolithic microfluidic chips show more and more limitations. A modular microfluidic system is a system composed of interconnected, independent modular microfluidic chips, which are easy to use, highly customizable, and on-site deployable. In this paper, the current forms of modular microfluidic systems are classified and studied. The popular fabrication techniques for modular blocks, the major application scenarios of modular microfluidics, and the limitations of modular techniques are also discussed. Lastly, this review provides prospects for the future direction of modular microfluidic technologies.

## 1. Introduction

After more than 30 years of development [1], microfluidic technology has made great progress in the fields of scientific research and industry. As a useful tool for researchers wishing to handle fluids of small volume, microfluidics has proven itself in various applications such as drug development/screening [2,3], tissue engineering [4], point-of-care testing [5], and chemical [6] and material synthesis [7,8]. In addition to academic research, microfluidic technologies have shown great potential in diverse industries and found use in various commercial devices such as inkjet printheads and digital polymerase chain reaction (dPCR) instruments [9]. However, despite the great progress that microfluidic technology has made, it is noteworthy that the development of a great number of microfluidic devices is still in the proof-of-concept and experimental stage [10]. This phenomenon is essentially caused by the widely used monolithic microfluidic instrument architecture, in which all functional components are integrated into a single microfluidic chip. On the one hand, monolithic microfluidic devices lack the flexibility to adapt to the constantly changing application scenarios. On the other hand, the design and use of the monolithic microfluidic system rely on peripheral equipment and expert operators. A highly flexible, field-deployable, and standardized microfluidic infrastructure would provide an easier way for the design and deployment of microfluidic systems, removing barriers to the further promotion of microfluidic technology.

In recent years, the concept of modular microfluidics has drawn great attention for advantages such as flexibility and ease of use. Equipped with customizable independent building blocks, modular microfluidics enables rapid and on-demand deployment of microfluidic systems, helping the popularization of microfluidic technology by providing a method of rapid prototyping and on-site reconfiguration. In this review, we discuss the current forms, applications, limitations, and future perspectives of modular microfluidic techniques.

So-called modular microfluidics refers to the integration of each separate function in microfluidics or micro total analysis systems (μTASs), in the form of independent microfluidic chip modules. In such systems, each module can be designed, machined, and tested separately, which improves the efficiency of system design and enables the system function to be reconfigured on demand, greatly expanding the application field of microfluidic technology. It is noteworthy that, in addition to modular microfluidic systems, there is another class of reconfigurable microfluidics, which dynamically change the routing or boundary of fluid channels in microfluidic devices through pneumatic actuation, phase transition, electrowetting, etc. [11], to modify the microfluidic structure and function in real-time. Such microfluidic devices have also shown great potential in field configuration and rapid deployment, but the reconfiguration is not based on module assembly; hence, it is beyond the scope of this paper.

**Figure 1:**
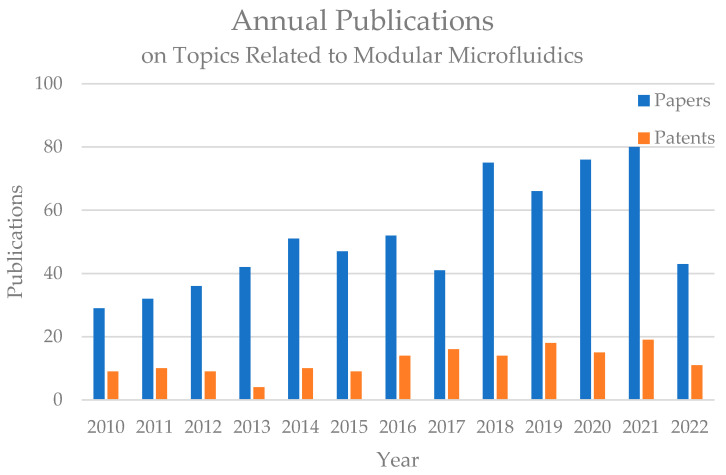
Annual publications on the topic of modular microfluidics. Paper search results were acquired from the Scopus Database, Elsevier with keywords ‘modular’ and ‘microfluidics’. Patent search results were acquired from the Patentscope database of the World Intellectual Property Organization (WIPO, Genève, Switzerland) using the search expression ‘FP (modular microfluidics)’.

Modular microfluidic technology has received increasing attention from the field recently. Figure 1 shows the annual research papers and patent publications on topics related to modular microfluidics between 2010 and 2022 according to the Scopus and Patentscope databases. The number of related studies on modular microfluidic technology has been increasing rapidly in recent years and has become a hot research topic, while patent applications related to modular microfluidics also exhibited substantial growth in this period, indicating that modular microfluidic technology is developing toward industrialization. In 2018, Fan et al. reviewed the development and applications of modular microfluidic technology [12]. With the rapid development of this field, in this paper, we summarize the current research on modular microfluidics, discuss the infrastructure and characteristics of different modular microfluidic systems, module fabrication techniques, and modularization-enabled application scenarios, and analyze the current challenges and future development trends of modular microfluidic technology.

## 2. Current Modularization Strategies

The most commonly used microfluidic systems are usually monolithic, which means the whole system has an all-in-one microfluidic chip with multiple integrated components, whereby each of the components has an independent job such as cell lysis or incubation. Therefore, the modification of a single function on-chip could mean redesigning, rebuilding, and retesting the entire chip. Considering how time-consuming and labor-intensive the fabrication process could be the monolithic design significantly lowers the efficiency of deployment. More importantly, in many circumstances such as point-of-care testing and public health emergencies, the monolithic design does not allow real-time decision-making on system design, making it inflexible and unpractical in such applications. Hence, there is a strong need to develop microfluidic systems that are readily available and field-deployable, and modular microfluidic technology is the key to meeting this need.

In this section, we discuss the forms and characteristics of previously reported modular microfluidic systems. Modular microfluidic systems reported are classified into the following categories according to the method of combining and aligning modules: multilayer stacking, backplate positioning, magnet-aided connection, electronics analogs, coaxial systems, shape complementarity, and threaded fitting. It is important to note that modular microfluidics in the broad sense should also include systems of multiple microfluidic chips interconnected by tubes, as is the case with commercial products offered by many companies. However, the modular systems constructed in such a way are not discussed in this section because of the large dead volume induced by the pipeline, the lack of convenience in module reconfiguration, and, most importantly, the inability to form a highly integrated, compact system.

### 2.1. Multilayer Stacking

Monolithic microfluidic chips are usually composed of two or several layers contained in a channel structure, and closed fluid channels are obtained through a bonding process [13,14,15]. Therefore, early modular microfluidic attempts were also based on the design of multilayer fluid channels, which divided the fluid channel structure belonging to different layers of a monolithic chip into independent microfluidic chips and obtained reconfigurable microfluidic systems by stacking multilayer microfluidic chips. For instance, Gonzalez proposed a modular assembly strategy of microsystems, where two silicon wafers as layered fluidic modules were interconnected through micromachined interlocking fins [16]. Grodzinski et al. proposed a modular system based on stacked polymer chips consisting of a mixer unit, an incubation unit, and a cell capture unit [17]. Since chips are no longer bonded to each other, each individual chip needs to have a closed fluid channel, and alignment and sealing must be considered when stacking chips between different layers. For this reason, the connection was realized by inserting barbed tubing into the connectors of chips containing rubber O-rings. Despite its convenience and reliability, such a stacked system still had a relatively large dead volume (39 μL) due to the barbed insertion.

**Figure 2:**
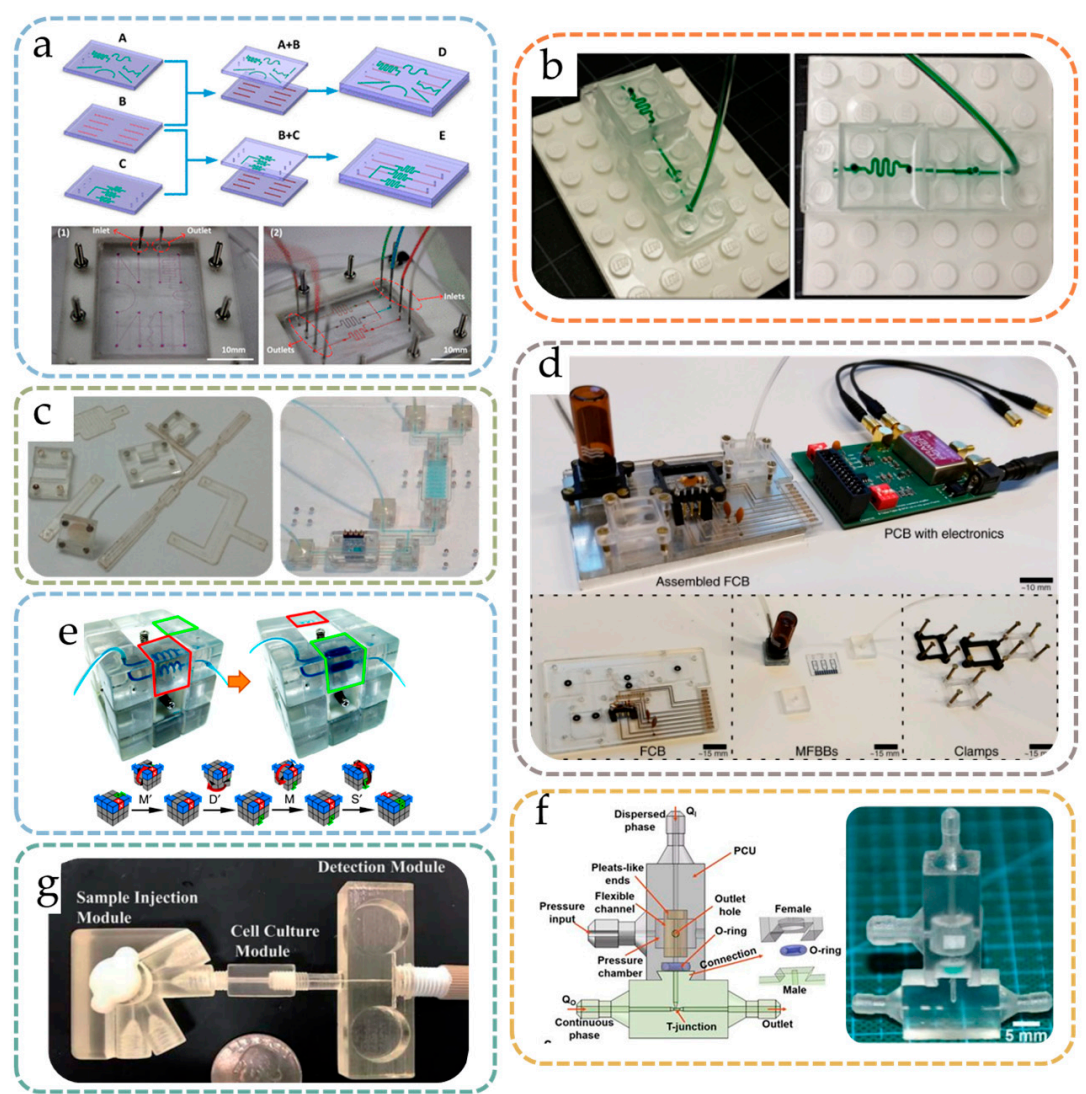
Different forms of modular microfluidic systems: (**a**) multilayered modular system with interchangeable layers by Qiu et al. Reprinted with permission from Ref. [18]. 2017, American Chemistry Society; (**b**) Lego^®^-style baseplate positioning of blocks by Vittayarukskul et al. Reprinted with permission from Ref. [25]. 2017, IOP Publishing; (**c**) magnet-enabled interconnection of microfluidics presented by Gomez et al. [31]; (**d**) microfluidic analog of a PCB, a fluidic circuit board with connectable module components, presented by Dekker et al. [37]; (**e**) Rubik’s cube-like coaxial microfluidic systems, where the microfluidic blocks rotate around three shared axes [41]; (**f**) modular assembly of interlocking blocks with shape complementarity, presented by Ji et al. [45]; (**c**–**f**) reprinted under Creative Commons Licenses; (**g**) threaded fitting of microfluid building blocks by Munshi et al. Reprinted with permission from Ref. [49], Royal Chemistry Society.

As PDMS became popular as a microfluidic chip material in past decades, its elasticity was found to be useful for nonpermanent bonding, which allows modification of one or several layers of a device. Qiu et al. proposed a modular microfluidic system for cell culture and biochemical analysis [18], where two layers of PDMS modules with open microfluidic channels fabricated using 3D-printed sacrificial materials were assembled into an integrated microfluidic system (Figure 2a). To prevent leakage, a clamping frame was used to immobilize the chips. Since the two layers of microfluidic channels were not permanently bonded, the function of the microfluidic system could be modified by simply loosening the frame and replacing the single-layer PDMS chips. Similarly, Mou et al. investigated a modular system based on a ‘hinge aligner’ [19], in which two prefabricated silicone elastomer chips with fluid channels were fitted together through a ‘flip chip’ holder that works like a hinge. The ‘hinge aligner’ is precisely CNC machined to ensure no relative displacement after the chip is embedded, and the alignment accuracy of the chip is up to 20 μm, making it easy to fit and align the chip.

For multilayer microfluidics, alignment between different layers is key to ensuring proper system function and preventing leakage. Many multilayer modular microfluidics have extra alignment mechanisms for the precise assembly of building blocks. Glick et al. devised a rapid assembly strategy for multilayered elastomeric microfluidic systems [20], where each layer was molded on both sides. In-mold alignment marks, which characterized the universal convex and concave geometry on each layer, were used to accurately position individual layers. Lee et al. proposed a multilayer fluidic system consisting of vertically stacked thermoplastic microfluidic devices [21], in which several auxiliary alignment strategies, including hemispherical pin-in-hole, hemispherical pin-in-slot, and plate–plate lap joint, were studied.

### 2.2. Backplate Positioning

Backplate positioning modularization strategies refer to the strategy based on a baseplate with a repetitive auxiliary positioning structure, through which various types of modules can be attached and fixed to the baseplate. A perfect example of such a strategy is the world-famous Lego^®^ toy (LEGO System A/S, Denmark), where each kind of different Lego block can be attached to the same baseplate. Inspired by Lego^®^, Yuen developed a ‘plug-n-play’ modular microfluidic system, SmartBuild [22], which consisted of a base plate, fluidic components (modules), and H-shaped microchannel inserts (connectors). By aligning the H-shaped inserts on the baseplate and connecting them with fluidic components through miniaturized male/female Luer fitting, a leak-free fluidic connection was achieved. SmartBuild can be easily reconfigured on demand and can also enable 3D fluid topology [23]. Hill et al. studied a modular microfluidic system based on a backplane design [24], in which each module called a ‘multifluidic evolutionary component’ (MEC) had a basic fluid function (e.g., valving, pumping, or mixing) and could be connected to a backplate with positioning holes to form a multifunctional system. Vittayarukskul et al. proposed a microfluidic system based on Truly Lego^®^-like modules [25], as shown in Figure 2b. The modules had the exact same look as Lego^®^ bricks; by fitting the chip modules onto the Lego^®^ baseplate, one could obtain function-customizable microfluidic systems. By using the same geometry as Lego^®^ bricks, the system inherits the property of free stacking of blocks. Owens et al. also proposed a modular microfluidic system based entirely on Lego^®^-like bricks [26]. Unlike Vittayarukskul et al.’s work, Owens et al. used injection molding to create modules of thermoplastic polymers, and microfluidic channels were created on these modules by micro-milling. Rubber O-rings were used to help seal the connections between modules. Compared with previous work, this method adopted hard chip materials and the strategy of reinforcing the connection with auxiliary Lego^®^ plates, thereby improving the stability of the system. For baseplate positioning, the reconfiguration of microfluidic modules can be rapidly achieved by simply re-plugging the modules on the baseplate, which is advantageous for end-users. However, the baseplate strategy may suffer from poor connection performance, and extra connection strategies may be necessary since the modules are only positioned on the baseplate with no reliable fixation between interconnecting modules.

### 2.3. Magnet-Aided Connection

In addition to the nesting of physical structures, modular microfluidic chips can be aligned and connected using magnetic forces. A magnet combined with rubber O-rings was first used in chip/tube interfaces to promote the convenience and reliability of connections [27]. Yuen proposed a microfluidic chip design based on magnetic links [28]. In this design, 3D printing was used to create serpentine channel modules and entrance/exit modules. Ring magnets were then embedded in the inlet and outlet ports of these modules and encapsulated with either rubber O-rings or PI tape, such that customized microfluidic systems could be achieved by combining various modules by stacking ports with different magnetic polarities. Furthermore, Ong et al. proposed a Tetris-style multiorgan simulation platform based on magnetic links [29], where ring magnets were also embedded at the in/outlet of blocks. Abhyankar et al. proposed a reversibly sealed, easily interchangeable, modular microfluid called SEAM [30], in which the magnetic attachment of blocks to housing could be coupled or uncoupled, simplifying cell seeding, culture, and downstream analysis procedures. Gomez et al. also proposed a modular microfluidic system based on magnetic connection [31], as shown in Figure 2c. Instead of using donut-like magnets, round magnets were embedded not directly into the chip, but into rigid plastic clamps that interconnected the microfluidic modules. The advantage of this approach is that there was no need to consider embedding magnets in the microfluidic chip, the chip was easy to process, and the dead volume induced by the inter-chip connection was small. Using modular microfluidic sensor modules, the authors demonstrated the on-chip sensing capabilities for pH, conductivity, and electroactivity. Generally, the magnet-based modular design has easier reconfiguration steps, but users have to pay attention to the reliability of the interconnection since the magnet may fail in certain conditions such as high temperature.

### 2.4. Electronics Analogs

Modularization is an important and common design idea in the electronics industry; hence, many modular microfluidic designs refer to modularization in circuits. For instance, Shaikh et al. proposed a modular microfluidic design based on a ‘fluid breadboard’ [32]. The main idea of the design was to divide the functions of fluid components into two categories: active and passive. Active components included a complex structure of active microfluidics devices, such as sensors (flow, temperature, pressure sensor, etc.) and actuators (pump, valve, active mixer, etc.), all of which were integrated into an ‘active’ microfluidic chip called a fluid breadboard. On the contrary, the ‘passive’ microfluidic channels and chambers were integrated into another ‘passive’ microfluidic chip. The two layers of chips were connected by silicon wafers with through-holes. When the configuration of the microfluidic system needs to be changed, the passive microfluidic chip (which is simple in structure) can be redesigned in a relatively simple way, without the need to redesign the whole microfluidic system from scratch, thereby simplifying the design and fabrication steps. Similarly, Skafte-Pedersen et al. investigated a modular system based on reversibly attached PDMS fluid control modules [33], which consisted of a soft active chip used for fluid control and a passive chip that could be rapidly batch-fabricated; the two chips were in reversible contact with each other through a clamp. This system can be renewed and reconfigured by simply replacing passive chips.

As for electronics analogs, another commonly employed modularization technique is the fluidic motherboard or the counterpart of printed circuit boards (PCBs) in microfluidics. On the fluidic circuit board, standardized interfaces or slots are reserved, and microfluidic chips as independent modules can be placed in the corresponding position. Perozziello et al. presented a modular microfluidic system based on a fluidic motherboard [34], where reversible PDMS sockets were used to position and connect different modules. Similarly, Brammer et al. proposed a modular optical microfluidic system based on a backplane, in which the backplane provided reversible fluid and optical connections for adjacent fluid units [35]. Chen et al. also designed a fluid motherboard for detecting bacterial pathogens [36]. Dekker et al. proposed a ‘fluid circuit board (FCB)’ modular microfluidic system similar to a printed circuit board [37], as shown in Figure 2d. In this system, functional components in microfluidics were made into standardized microfluidic modules with uniform specifications and connected to an FCB via a flow channel using special clamps. The authors pointed out that the standardized design can bring convenience to the development of highly customized point-of-care testing (POCT) equipment.

The similarities and differences between the motherboard strategy and baseplate strategy should be noted; both are important for module assembly, but the former contains fluid channels in its motherboard for fluid routing, while the baseplate of the latter does not contain fluid channels and is only used for block positioning.

### 2.5. Coaxial Systems

The coaxial system denotes a microfluidic system in which some functional modules are interconnected by nesting each other with common surfaces, usually using an interference fit. The advantage of such coaxial systems is that many devices (capillaries, sensors, etc.) and processes (drilling, turning, etc.) produce natural cylindrical surfaces, which is convenient for the alignment and interconnection of devices. Bhargava et al. reported a modular microfluidic system based on Lego^®^-like bricks [38], in which all the different microfluidics modules had a similar appearance, similar to a 1 × 1 Lego^®^ block part, but the flow channel structures inside modules varied, and the modules were connected coaxially by an interference fit of cubic male/female connectors. Functional microfluidic systems such as mixers and droplet generators can be obtained by combining microfluidic modules with different flow channels. This method has the advantages of convenience in use and 3D fluid topology, but the microfluidic modules used are uniform in size, and a large number of microfluidic modules are needed to construct complex microfluidic systems. Cronin et al. proposed the concept of ‘programmable’ microfluidics [39], also using 3D printing technology to fabricate microfluidics chip modules, and they designed Lego^®^-inspired male and female connectors for these microfluidics modules. By combining these microfluidic modules, a microfluidic system with mixing, droplet generation, fluid control, and sensing functions can be obtained. The modular method has greater flexibility because it has no restriction on chip shape. However, the low precision of the extrusion molding technology used in fabrication and the resulting rough surface can lead to leakage between chips, which limits the practicality of the technology. Bandulasena et al. proposed a modular microfluidic system based on capillaries [40], where micron-scale capillaries could be aligned accurately by micro-milled holes and Lego^®^-like docking structures. For most coaxial systems, the simple interference fit between assembly blocks enables the easier design of the blocks, but the performance of the interconnections is highly dependent on the quality of contacting surfaces of the blocks. Unlike using interference fit, Lai et al. proposed a modular microfluidic system based on the Rubik’s cube [41], in which the microfluidic modules are integrated into the system as parts of the Rubik’s cube, as shown in Figure 2e. There are three main components in the Rubik’s cube system: corner blocks, edge blocks, and center blocks. The corner blocks and edge blocks contain fluid channels that can be freely combined with each other, while the center block is responsible for providing all blocks with pushing force from a spring to the center of the Rubik’s cube, allowing adjacent blocks to fit closely. Because the connection and alignment of modules depend on the fit of a common cylindrical surface and rotation around fixed axes, the system can also be regarded as a coaxial system.

### 2.6. Shape Complementarity

The complementarity of shapes can be used to make reliable connections between objects, such as dovetail joints and jigsaw puzzles. Rhee et al. proposed a microfluidic chip design and fabrication method based on microfluidic assembly blocks [42]. The core idea of this process is that the end-user can design microfluidic systems by pasting the modules on substrates using adhesives and pre-molded PDMS microfluidic chips as basic modules. Compared with the traditional microfluidic chip processing method, this technique has the advantages of less manpower and time consumption, simple operation, and low-expertise requirement. The fabrication method of the microfluidic chip was further improved by adding a self-alignment mechanism to the microfluidic assembly block [43]. Since the alignment and sealing between modules are similar to a jigsaw puzzle, this method enables the application scenario of on-site assembly of microfluidic modules, but it lacks sufficient flexibility due to the permanent bonding, which makes the microfluidic system unchangeable once assembly is complete. Lee et al. proposed a module alignment method based on shape complementarity [44], in which microfluidic modules had cone-shaped bumps and depressions on the inlet/outlet surface. When connecting modules, bumps and depressions between adjacent modules were filled with each other to ensure alignment between modules. At the same time, O-rings and metal pins were used to strengthen the connection and prevent leakage. Ji et al. proposed a modular microfluidic system for the generation of multiple emulsion droplets [45]. The system also utilized a dovetail-style modular connection, with rubber O-rings embedded between microfluidic modules (Figure 2f). Unlike Rhee et al.’s proposal, the modular microfluidic system did not require additional reinforcement after assembly and could tolerate pressure up to 4 bar through a direct combination of the blocks. Song et al. [46] also designed a modular microfluidic system for droplet generation based on Lego^®^-like interconnections. However, the difference is that the design of the male and female connector added a shape-complementarity linkage mechanism. After the male connector is plugged into the female connector, the protrusion on the male connector can fit into a track of the female connector, preventing leakage at the joint after rotation; thus, high working pressure can be achieved. An important advantage of the shape complementarity strategy is that the shape complementarity of modules enables automatic alignment or tightening of blocks after assembly, which is advantageous for end-users.

### 2.7. Threaded Fitting

Threaded fitting can be considered a special case of a coaxial system, in which a pair of interconnected microfluidic modules have internal and external threads, which ensure the reliability of the connection. Due to the need to process the internal and external threads while constructing the fluid channel, the threaded fitting blocks are mostly created by 3D printing. Zhou et al. proposed a modular emulsion generation device based on 3D printing [47]. By combining the continuous phase supply module and dispersed phase supply module using a thread and capillary, the device successfully generated single emulsions, Janus emulsions, and double emulsions. Similarly, Morimoto et al. proposed a coaxial microfluidic system based on a threaded fitting [48], which could be used for the selective generation of single or dual emulsions and double-layer or triple-layer fibers by changing the type and number of modules used.

One benefit of threaded fitting is that it can be combined with commonly used, commercially available fluid components. For example, Munshi et al. [49] proposed a threaded connection-based modular system in which threads could be used not only for the connection of various functional modules but also to attach commercially available finger-tight fitting products, as shown in Figure 2g. The plug-and-play modular microfluidic system proposed by Zhang et al. [50] and the modular interface proposed by Maillard et al. [51] also employ threaded fitting blocks that can be connected with finger-tight fitting to facilitate the connection of the device with external tubing.

## 3. Manufacturability of Microfluidic Modules

Modular microfluidics relies on discrete building blocks that are designed to perform various functions, and many of these blocks should have complex or 3D geometry to enable the easy assembly of modules. For instance, Lego-like modular microfluidics requires a millimeter-scale extrusion/concave region to be constructed on the blocks, magnet-based blocks need magnets to be embedded, and Rubik’s cube-like modular systems need rubber O-rings to be embedded on the modular elements. Therefore, ordinary fabrication techniques such as wet/dry etching, thermal bonding, and basic soft lithography are no longer the primary choice for processing these irregular microfluidic chips. To address this, novel fabrication techniques are widely employed in processing these modular microfluidic blocks. These fabrication methods can be generally divided into two categories: additive manufacturing methods and subtractive manufacturing methods. Each method for the customization of microfluidic blocks has its merits and limitations, which should be carefully investigated before designing a modular system. In this section, we discuss the manufacturability of different modular microfluidic systems from the perspective of manufacturing techniques.

### 3.1. Additive Manufacturing

**Figure 3:**
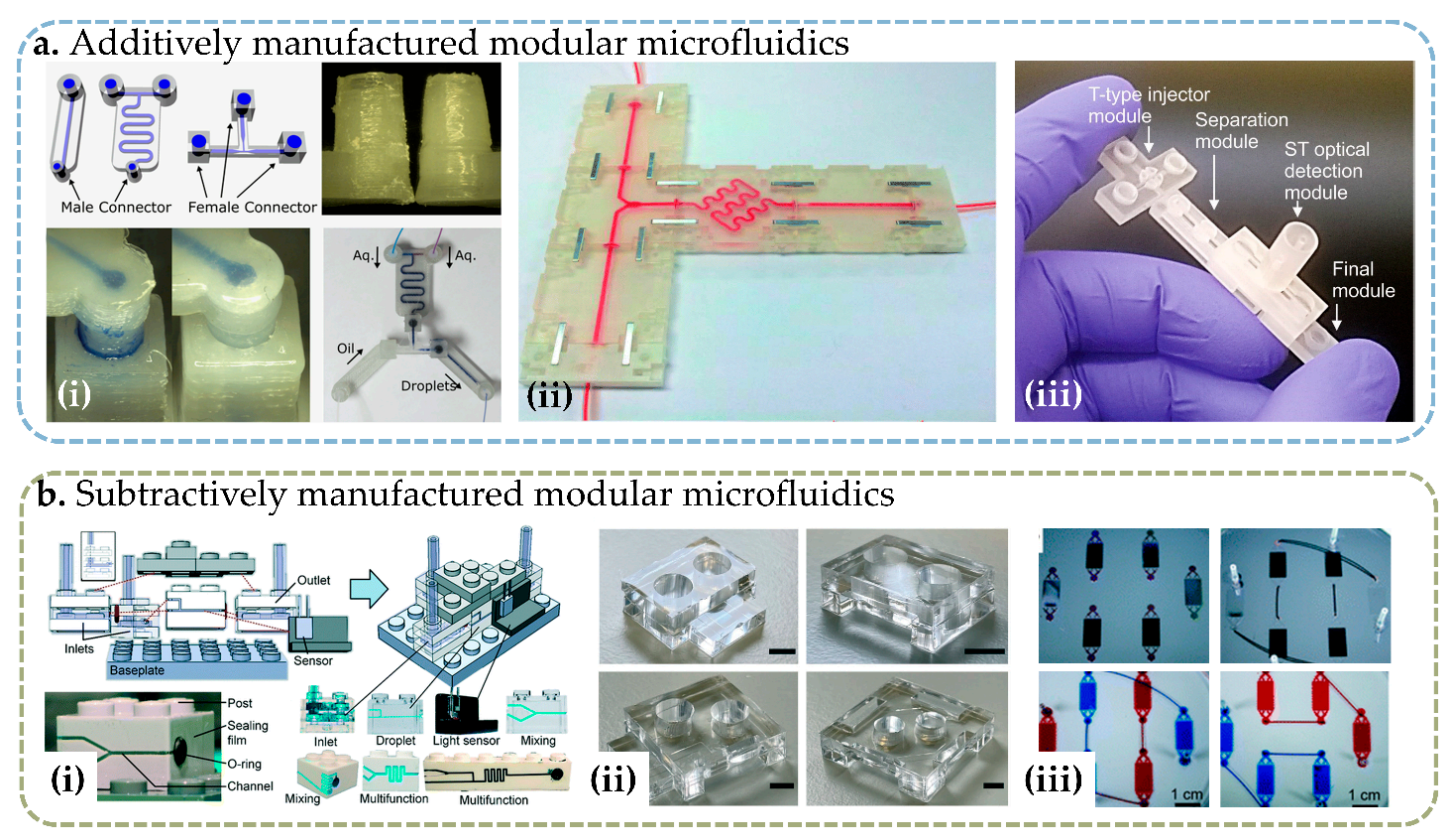
Photographs of modular microfluidic assembly blocks fabricated through different techniques: (**a**) microfluidic blocks created by additive manufacturing, including (**i**) FDM 3D-printed blocks [39], reprinted under Creative Commons CC-BY license, (**ii**) SL 3D-printed microfluidic assembly blocks, reprinted with permissions from Ref. [44]. 2014, Royal Society of Chemistry. (**iii**) PolyJet 3D-printed blocks, reprinted from Ref. [63]. 2019, IOP Publishing; (**b**) microfluidic blocks created by subtractive manufacturing, including (**i**) micro-milled Lego-like modules on injection-molded blocks [26], (**ii**) microfluidic blocks created by laser ablation [68], and (**iii**) microfluidic blocks created using template molding on a lithographic patterned and reactive ion-etched templates [69]. Reprinted with permissions from the Royal Society of Chemistry.

Additive manufacturing, also known as 3D printing, is a technique to deposit material layer by layer on a detachable or nondetachable substrate. Additive manufacturing allows the fabrication of devices of arbitrary shape; thus, it is especially suitable for designing microfluidic building blocks with an irregular shape, as shown in Figure 3a. Since there are already excellent reviews on the 3D printing of microfluidic devices [52,53,54,55], this paper focuses specifically on the 3D printing of modular microfluidic building blocks.

#### 3.1.1. Fused Deposition Modeling (FDM)

FDM is the most common extrusion-based 3D printing technology, which uses a movable extruder head to add material in a layer-by-layer manner to previously processed material. In FDM, the material is melted in a nozzle and then extruded onto the previous layer, where the new material is fused with the previous layer before cooling. The biggest advantage of FDM technology is its low cost and its capability of processing a large variety of common thermoplastic polymers including acrylonitrile–butadiene–styrene (ABS), polylactic acid (PLA), and polycarbonate (PC) [53]. In addition, FDM technology can use composite materials and multiple materials. In reported work, FDM printing is reported to create Lego^®^-like blocks [39] and capillary-driven bricks [56], magnetic blocks [28], and sacrificial templates [57] of modular devices. Due to its low cost, rapid processing capability, and simple operation, FDM technology plays an important role in many microfluidic applications [58]. However, FDM technology has many obvious disadvantages in microfluidic chip fabrication. For example, FDM printing typically has submillimeter accuracy, produces structures with coarse features and rough surfaces [59], and is more prone to deformation and damage due to internal stress caused by high-temperature processes, setting limitations to its applications in modular assembly.

#### 3.1.2. Stereolithography (SL)

SL is another mainstream 3D printing processing technology, which uses optical methods to selectively cure photosensitive resin materials layer by layer. Compared with FDM, SL technology is more suitable for the rapid construction of fine structural features. The curing range of photosensitive resin is controlled layer by layer using illumination to realize the processing of the 3D structure. SL technology is widely used in modular microfluidic devices; it can be used to directly create voids in microfluidic building blocks [38,41], but it can also process microfluidic chip molds [20]. Recent studies have shown that customized desktop-grade SL 3D printers can achieve microchannels with minimum sizes as low as 18 × 20 μm [60], sufficient to support the vast majority of microfluidic applications. However, SL has its drawbacks; unlike FDM, SL can print a limited variety of materials, while it is difficult to print multiple materials at the same time or to use sacrificial materials to build large cavities.

#### 3.1.3. Photopolymer Inkjet 3D Printing

Photopolymer inkjet 3D printing, or PolyJet, is an emerging technology through which the photosensitive resin ink is accurately sprayed on the part to be printed, and the light source on the print head is used to illuminate the ink to cure the resin and form a three-dimensional structure. This method uses photosensitive resin similar to SL [61], which has high theoretical accuracy and can use multiple materials in a single device [62]; therefore, it can also be used to process different kinds of microfluidic blocks [46,49,63]. In addition, hollow structures can be released by using special soluble ink as sacrificial materials. The disadvantages of PolyJet printing technology are that the printer is expensive, and it must be used with special ink materials.

### 3.2. Subtractive Manufacturing

As opposed to additive manufacturing, subtractive manufacturing refers to the method of removing material from a bulk substrate. Typical subtractive manufacturing techniques include dry/wet etching, micro-milling, and laser ablation. Subtractive manufacturing is commonly used for the fabrication of monolithic microfluidic chips, but it can also be used for the fabrication of microfluidic building blocks with proper adaption (Figure 3b).

#### 3.2.1. Micro-Milling

Micro-milling is a subtractive manufacturing technology that uses a rotating endmill to remove material from the bulk substrate [64]. With the advancement of technology, the existing CNC machine tools can achieve positioning accuracy up to 1 μm, and they can directly process arbitrary fluid channels and cavities (the minimum feature size is related to the cutter head size). Owens et al. used micro-milling to process fluid channels on injection-molded Lego^®^ blocks [26] and compared them with FDM 3D-printed parts, demonstrating a higher accuracy and better alignment performance of micro-milling. Micro-milling is an ideal method for rapid prototyping design. Although high-precision CNC machine tools are expensive, they can provide better machining accuracy and less deformation; thus, they can be used in occasions requiring higher performance of alignment and connection [40,65].

#### 3.2.2. Laser Ablation

The high power of the laser makes it possible to directly ablate or cut microscale patterns. In terms of processing microfluidic building blocks, Marquez et al. used a CO_2_ laser to ablate PMMA microfluidic chips for multilayered assembly [66]. Gerber et al. created laser-cut microfluidic building blocks for education and fast prototyping [67]. In addition to directly etching fluid channels, laser ablation can be used in machining housing or clamps for module alignment [30], as well as the external shape of an entire building block [68]. The equipment used in laser ablation is usually not very expensive, but the method may cause melted edges and deformation when ablating polymers, which may negatively affect channel structure and assembly accuracy.

#### 3.2.3. Polymer Processes with Subtractive Manufactured Templates

One can also use molding techniques to create microfluidic modules, with the help of a template made by subtractive manufacturing. For example, thermosetting polymers such as PDMS elastomer can be cured on a template made through a photolithographic process, widely known as soft lithography, which is used in modular microfluidics [69,70]. For structures that cannot be directly molded by soft lithography, such as male/female connectors for module alignment and joining, elastomer casting can be performed using a specially designed container mold [25,71].

For microfluidic modules composed of thermoplastics, the microfluidic structures can be formed by pressing the polymer into a precisely defined solid mold after heating the polymer beyond the vitrification temperature. Common techniques for thermoplastic polymer chip fabrication include injection molding, hot embossing, and laminating [72], which can, of course, also be used in fabricating microfluidic assembly blocks [73,74]. In thermoplastic molding, the mold or template can be prepared by subtractive manufacturing methods such as RIE and micro-milling. After molding, it is generally necessary to seal microfluidic channels with thermal bonding or adhesive bonding [75]. Despite the high cost of preparing precision molds, the thermoplastic molding technique usually has very high efficiency and allows rapid mass production of thousands of identical devices, making it the most attractive processing method for commercially promising microfluidic modules [76].

To conclude, Table 1 provides a comparison of the performance of different manufacturing techniques and their applicability in modular microfluidics. Generally, the additive manufacturing techniques are more adaptive due to the capability of creating arbitrarily shaped devices; protrusions, concaves, and structures for embedding rubber O-rings and magnets can be easily created on the microfluidic blocks. Thus, additive manufacturing techniques can be used in the fabrication of almost all kinds of modular assembly blocks, representing the dominant approach for modular microfluidics. The major problem of additive methods is that the limited choice of chip materials (thermoplastics or thermoset resins) prevents the use of devices in many applications. On the other hand, subtractive approaches such as laser ablation and template molding allow the processing of a larger variety of materials, but they cannot directly create 3D structures for the interconnects of modules. When selecting a manufacturing method for microfluidic modules, the specific requirements of the application (e.g., channel size, air permeability, pressure tolerance, and solvent compatibility) need to be fully considered; thus, a hybrid process may be necessary for satisfactory devices.

## 4. Applications Scenarios of Modular Microfluidics

Due to its ease of configuration and capability of field deployment, modular microfluidics has a large variety of applications. Instead of introducing specific applications, here, we focus on the classification of different scenarios that many applications share in common, and we discuss how modular microfluidics is useful and attractive in such application scenarios.

### 4.1. Modular Microfluidics for Ease of Design

**Figure 4:**
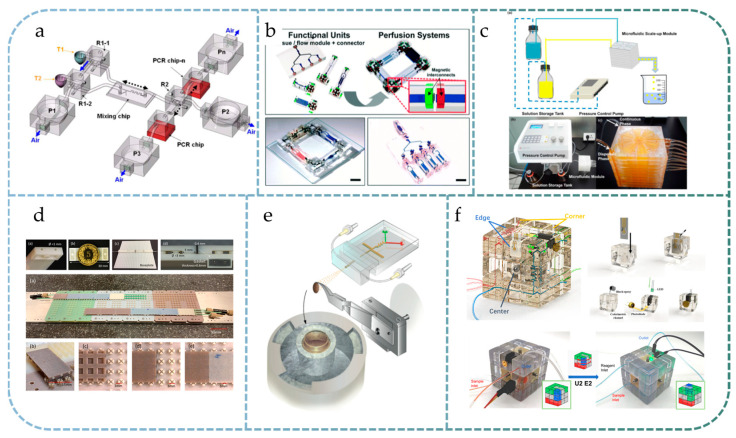
Photographs of modular microfluidic systems for different application scenarios: (**a**) modular microfluidic design for on-chip PCR system that reduces the risk of a single point of failure, reprinted with permission from Ref. [79]. 2011, Springer Nature; (**b**) modular building blocks to study the interactions between multiple organs-on-chips, Reprinted with permissions from Ref. [29]. 2019, Royal Society of Chemistry.; (**c**) modular microfluidics for highly parallel reactions of microsphere synthesis by stacking 10 identical modules. Reprinted with permission from Ref. [83]. 2018, IOP Publishing; (**d**) modular microfluidics for unlimited extension of cooling substrates used in high-energy physics detectors. Reprinted with permission from Ref. [86] under Creative Commons CC-CY license; (**e**) modular design of a microfluidic system for parameter control in cryo-EM sample preparation, Reprinted from Ref. [89] under Creative Commons CC-CY license; (**f**) Rubik’s cube-like multifunctional modular microfluidic system as a reconfigurable analytical instrument that allows on-site deployment of a sensing platform for analysis of different water pollutants. Reprinted with permission from Ref. [101]. 2021, IEEE.

Modular assembly can reduce the design and machining costs of microfluidic systems, improve the efficiency of the process from draft to final product, and save time and manpower. These advantages are reflected in almost all modular systems. For instance, Godino et al. proposed a three-layer structure of a modular microfluidic system [78] facilitating magnetic capture and electrochemical detection. In this system, each function module can be replaced and reused arbitrarily, which improves the flexibility of the system and saves the overall cost. Liou et al. proposed a modular microfluidic design for portable microfluidic analytical devices [79] and established a PCR platform for DNA amplification using three basic components: pumps, valves, and reservoirs (Figure 4a). The authors pointed out that the modular design could reduce the risk of rework of the whole system due to a single point of failure, as well as reduce the cost of trial and error. Hao et al. studied a modular microfluidic system for mass spectrometry [80]. The semiautomatic detection of insecticides and insecticide metabolites was achieved by serial connection of the pretreatment module, gas chromatography separation module, and nano-electrospray ionization module, avoiding a tedious manual operation process.

### 4.2. Modular Microfluidics for Arbitrary Combination

An important application scenario for modular microfluidic systems is where many different module combinations need to be tested. For example, in the study of organs-on-chips, researchers are often interested in studying the interactions between multiple organs/tissues [81]. Modular microfluidic technology can provide convenience for studying an arbitrary combination of different organ chips. In [29], Ong et al. proposed a modular microfluidic platform named the Self-Aligning Tetris-Like modular microfluidic platform (Figure 4b), in which magnets and PDMS microfluidic modules were cast into a 3D-printed scaffold, and the excellent permeability and biocompatibility of PDMS materials were utilized to enable an on-chip culture of human organ tissues. Using this modular design, researchers can easily construct microfluidic systems that contain multiple human organs and tissues. The system also includes a gas-driven peristaltic pump module that allows fluid circulation and perfusion between multiple modules, more realistically reflecting the interactions between multiple organs. The authors demonstrated applications in constructing recirculating multiorgan systems to emulate liver-mediated bioactivation of nutraceuticals and prodrugs to modulate their therapeutic efficacies in the context of atherosclerosis and cancer. In [65], Yu et al. proposed a reconfigurable microfluidic cell culture system that enables tissue modeling via the three-dimensional free assembly of stacked layers containing predefined microenvironments. Through simple assembling and disassembling of the stacks, spatial and temporal maneuverability for 2D and 3D analysis of multiple cell types can be achieved.

### 4.3. Modular Microfluidics for High Parallelization

The use of modular microfluidics enables the stacking of large, repetitive, or similar functional modules in a single microfluidics system, making it suitable for situations where a large number of parallel tests are required. For example, Li et al. designed a multiple modular microfluidic (M^3^) reactor [82] with a droplet generator and a polymerization chamber based on a modular design (Figure 4c), which can manipulate the number of simultaneous polymerization reactions by linking parallel modules on demand, thus achieving optimized productivity. For microsphere synthesis, Huang et al. studied the design criteria for stacked modular systems for parallel scaling [83]. By stacking the same module over ten layers, they obtained increased microsphere synthesis efficiency and improved CV, proving that modular design can be used for high-throughput applications. Yue et al. proposed a large-scale perfusable microvascular network based on stacked microfluidic modules [84]. The network could be easily extended to a large scale by stacking tissue chambers and medium channels, demonstrating the system’s scalability, which is essential for a wide range of multiorgan-on-a-chip applications. Jones et al. presented a modular microfluidics-based endothelial model [85]. Owing to the scalability of modular microfluidics, the presented endothelial model can be massively produced to perform multiple cellular analyses in a parallel manner.

### 4.4. Modular Microfluidics for Unlimited Extension

In addition to parallel scaling, another advantage of modular microfluidic technology is a serial extension, whereby it is easy to extend the modular system from a basic case to more complex applications, such as the capillary electrophoresis system based on modular microfluidics studied by Walczak et al. [63]. In this system, the length of the electrophoresis channel can be easily extended according to the need, such that the length of the separation channel is no longer limited by the size of a single device. Moreover, the modular design enables transferring of ‘frozen’ samples among different microfluidic systems. Angeletti et al. at CERN proposed a modular cooling system for sensors used in the high-energy physics field, named the ‘Interlocking Modular Microfluidic Cooling Substrate’ (i-MμCS), shown in Figure 4d [86]. The system uses a Lego^®^-style baseplate for positioning, on which fluid channel modules as cooling substrates are linked together through gaskets. The size of such a system expands freely according to the actual needs; therefore, it can be used for cooling silicon detectors applied in high-energy physics. Frische et al. proposed a system called Microfluidic Stack [87], in which layers have a modular design and are linked by permanent bonding. The authors emphasized that the system can be extended by easily adding new modules.

### 4.5. Modular Microfluidics for Parameter Control

For some applications, it is necessary to control one or more parameters in microfluidic system design and study the influence of different parameters on test results. For such applications, it could be cumbersome and impractical to design and manufacture different monolithic microfluidic chips for each parameter combination, while modular microfluidic chips can provide an easy method for parameter control. For example, in many modular droplet microfluidic systems, the droplet generation process can be manually adjusted by changing the design parameters of specific individual modules [88]. Maeots et al. proposed a modular microfluidic system for cryo-electron microscopy sample preparation, as shown in Figure 4e [89]. Due to the difficulty of short-lived intermediate enrichment in biochemical reactions, a modular microfluidic assembly consisting of a mixing-incubation chip and a nozzle chip was employed, where the length of the transmission line in the mixing chip could be used to control incubation time precisely. A series of modular microfluidic devices with different residence times of 10, 20, 80, 200, 400, 480, 800, and 1330 ms were fabricated, demonstrating the time control capability of the proposed method. Abdel-Latif et al. presented a facile room-temperature, single-solvent strategy for continuous bandgap tuning of colloidal perovskite QDs through a modular microfluidic platform, QDExer [90]. The modular design of the microfluidic platform enables studying the effects of ligand composition and halide salt source on the rate and extent of the halide exchange reactions.

### 4.6. Modular Microfluidics for On-Demand Reconfiguration and Instrumentation

For a pure modular microfluidic system consisting solely of microfluidic channels and chambers, the operation of the system requires the use of external actuators (pumps and valves) for execution and external sensing elements for data acquisition, making the system incomplete and non-independent. Actually, in a broad sense, modular microfluidic systems should also include actuator modules (pumps, valves, heating, and oscillators) and sensor modules (optics, impedance, and electrochemistry) in addition to flow modules to form an independent, self-sufficient instrument system with execution and sensing capabilities. Therefore, independent modules for standalone functions such as debubbling [91,92], mixing [93], pumping [93], microwaving [94], temperature control [95], heat exchange [96], cell culturing [97], spectrophotometry [98], pathogen detection [99], and immunoassay [100] have also been studied for integration into versatile microfluidic systems.

For portable analytical instruments such as POCT equipment, a modular design can improve the flexibility and ease of use of the system and reduce the time required for system implementation. Therefore, a microfluidic system equipped with various modules (either fluidic modules or nonfluidic modules) shows good field deployment capability and ease of use, and it can be used as the platform for intelligent analytical instruments. For example, Lai et al. proposed a functional reconfigurable multifunctional sensor system based on the Rubik’s cube [101], as shown in Figure 4f. By integrating the electrochemical analysis module and colorimetric module in the microfluidic cube, a multifunctional field-deployable instrument was created, allowing on-site reconfiguration for the detection of multiple water pollutants. Glide et al. proposed a modular design for microfluidic instruments called MATAS [102], which uses a PCB as a universal housing for both electric and flow circuits. Electronic modules (sensors, actuators, etc.) are assembled on one side of the PCB, with flow path components on the other. The user can freely assemble each module as needed, obtaining synthetic/analytical instruments with custom functions. Similarly, Hill et al. [24], Sabourin et al. [103], Dekker et al. [37,104,105], Gomez et al. [31,106], and Lee et al. [107] proposed reconfigurable instrument architectures based on modular microfluidics. Although the implementation forms and demonstrative applications may differ, these efforts have a starting point in common: to achieve portable, customizable, field-deployable, general-purpose, and automated instruments through modularization, reducing the time consumption and expertise dependence in building experimental platforms.

## 5. Challenges of Modular Microfluidics

### 5.1. Arbitrariness of Connection

In many modular microfluidic systems, the designers claim that the assembly blocks can be connected arbitrarily on demand. However, most systems have restrictions on the connection of modules, such as the need to distinguish between male and female connectors [49], convex or concave geometry [45], the polarity of magnets at the joints [28], and whether there are O-rings embedded [41]. Therefore, the connection between modules is not arbitrary but needs to meet some preset rules. Since there is no real arbitrariness in building fully custom microfluidic systems using these modular blocks, there is still a probability that users will need to redesign modules for customized applications rather than using off-the-shelf assembly blocks, hindering the practical use of the modular system.

### 5.2. Dead Volume and Resulting Waste

Dead volume is an important problem for modular microfluidic systems compared with monolithic systems. The monolithic system, due to its unique compactness, can operate with minimal reagent and sample loss. Although modular microfluidics saves dead volume compared to multichip systems with tube connections, this problem inevitably exists because of the interconnections between modules. The use of more compact connections, such as narrower fluid channels and smaller O-rings, can further reduce dead volume waste, but may simultaneously set challenges for the alignment strategy of the modules.

### 5.3. Configuration Convenience

The ease of configuration is an advantage of many modular microfluidic systems. In contrast to monolithic microfluidic systems, modular systems do not require repeated microfluidic chip manufacturing; hence, the deployment of the system can be achieved by non-specialists at the site of deployment. However, for those microfluidic modules that are easy to assemble and reconfigure, such as those imitating Lego^®^ bricks [25], the connections lack adequate fastening mechanisms between modules, and the reliability of inter-module connections is weak; therefore, they cannot be used in complex environments such as high pressure. Reliable connections, such as the use of clamps with fastening threads [18], will lead to a complex and time-consuming chip reconfiguration, thus hindering rapid or real-time reconfiguration of functions. Balancing easier reconfiguration with higher system reliability is an important issue that many modular microfluidic technologies need to address.

### 5.4. Material and Process Limitations

As mentioned earlier, fabrication techniques, as well as the materials that can be used, set limitations for modular microfluidic systems. When using a modular microfluidic system, the fabrication processes and materials used make specific applications unsuitable. For example, many modular microfluidic systems use SL 3D-printed photopolymer resins, which result in inadequate air permeability, thereby preventing the survival of cells and tissues in long-term culture. The thermoplastic microfluidic modules created by FDM 3D printing should avoid using a liquid containing acetone, chloroform, or specific organic solvents; otherwise, this may lead to chip dissolution and adhesion. Some microfluidic systems use molded silicone elastomer chips, whose hydrophobic and porous properties may lead to adsorption of reagents and swelling in the presence of organic solvents [108]. Some modular systems use permanent magnets for the assembly of blocks; accordingly, they should not be used at high temperatures to avoid magnet failure. Therefore, when designing modular microfluidic systems for specific applications, full consideration should be given to the fabrication techniques and materials.

## 6. Conclusions and Outlook

Modularized microfluidics is an interdisciplinary field involving physics, biology, medicine, materials, engineering, etc. The existing research work fully demonstrates the great potential of modular microfluidic technology. This paper aimed to introduce the development status of modular microfluidic technology, as well as summarize the modularization forms, fabrication technologies, and applications of modular microfluidics. In the future, modular microfluidic technology is expected to advance in (but not limited to) the following areas:

- In terms of the breadth of modular microfluidic systems, innovative forms of modular microfluidic systems can be further studied, such as paper-based modular microfluidics [109,110], digital modular microfluidics [111], template modularity-based microfluidics [112], and improved inter-module connections using advanced materials such as self-healing hydrogels [113,114] and superhydrophobic materials [115];
- In terms of the depth of modular microfluidic techniques, by tracking the latest advancement in technology, the use of novel processing techniques and chip materials should further improve the aligning accuracy and performance of modular systems, expanding the range of available modular design. At the same time, the arbitrariness of module connection should be further improved (such as eliminating the need to distinguish between male and female connectors), improving the performance and ease of use of the modular microfluidic systems;
- In terms of applications, versatile modular blocks with sensors and actuators should be implemented for further exploration of meaningful modular microfluidic applications, e.g., replacing currently used nonportable, expertise-requiring analytical instruments with fully modular, portable microfluidic equipment for easy setup of point-of-care testing or rapid response to public health emergencies;
- In the aspect of fundamental research, methods and theories for on-demand reconfiguration of the modular blocks and improvement of the performance of modular interconnections should be studied or adapted from other fields, which will be beneficial for general microfluidic systems;
• In terms of industrialization, academia should work closely with industry to develop common standards for modular microfluidic systems while developing reliable and easy-to-use modular microfluidic systems to facilitate the dissemination and implementation of technologies.

In the future, with the help of modularization, microfluidic technology is expected to be more widely used. The authors of this paper envisage a future modular microfluidic system in a form where a microfluidics device factory can fabricate interchangeable microfluidics modules according to accepted standards, end-users can purchase off-the-shelf modules to form highly compact modular systems without any expertise in device fabrication, and the modular system can be simulated and programmed through a universal and open-source software framework (just like electronic systems) to achieve the specific microfluidic system function. On the basis of the above conditions, users can obtain customized microfluidic systems, such as POCT devices or pollutant analysis devices, in a significantly reduced time, which can greatly speed up the design and deployment process of microfluidic systems, thus saving material and time. In order to achieve such a system, it is necessary to establish consensus-based microfluidics standards. Although vendors such as ChipShop, Dolomite, and Micronit all offer their own modular microfluidic products, they do not share a uniform standard, limiting connectivity compactness and chip interoperability. Fortunately, the community has long recognized the importance of microfluidic standardization, and many studies have attempted microfluidic standardization [116,117]. Earlier this year, the ISO approved the ISO 22916:2022 standard, which specifies the basic requirements for dimensions, connections, and initial device classification for the seamless integration of microfluidic components and facilitates the process of designing new microfluidic devices. Nevertheless, to build a fully interchangeable, compact, and easy-to-use modular microfluidic platform, substantial standardization work is still required [118].

Although modular microfluidic technology is never short of impressive advances, we believe that the development of modular microfluidics is still in the preliminary stage, and there remain many, many challenging topics to explore. As architectures continue to advance, manufacturing processes continue to be optimized, and applications continue to grow, we believe that modular microfluidics, as a platform tool capable of free customization, rapid reconfiguration, and field deployment, will achieve faster and better development in all fields in the near future.

## Figures and Tables

**Table 1:** Comparison of different manufacturing techniques for microfluidic modules.

	Manufacturing Methods	Feature (Void) Size [77]	Surface Roughness	Applicability in Modular Design	Advantages	Limitations
**Additive** **manufacturing**	FDM	>300 μm	Coarse	High (arbitrary external shape for interconnections)	Broad range of thermoplastics available Low cost	Coarse features and resulting leakage
	SL	Usually >150 μm	Medium (fine on forming plane)		Low cost	Limited range of materials
	PolyJet	>200 μm	Fine		Fine features	Limited range of materials High cost for facilities
**Subtractive** **manufacturing**	Micro-milling	>50 μm (dependent on endmill size)	Fine	Medium (allows 3D external shape)	Fine features Wide range of materials available	High cost for facilities
	Laser ablation	>50 μm	Coarse on edges	Low (inapplicable for complex external shape)	Fast processing	Coarse features on cut edges High cost for facilities
	Subtractive mold-based manufacturing	>5 μm (dependent on template)	Fine		Low cost per chip for batch production	Complexity in process Requirement for expertise and facilities
